# Supplementary material for: A gain-of-function TPC2 variant R210C increases affinity to PI(3,5)P2 and causes lysosome acidification and hypopigmentation
Source: Nat Commun. 2023 Jan 14;14:226. doi: 10.1038/s41467-023-35786-9 (PMC9840614; doi:10.1038/s41467-023-35786-9)
Supplement: Supplementary file 1 — Supplementary Information [file 41467_2023_35786_MOESM1_ESM.pdf]

A Gain-of-Function TPC2 Variant R210C Increases Affinity to PI(3,5)P_2 and Causes Lysosome Acidification and Hypopigmentation

Wang Q, et al.

Supplementary Figures and Figure Legends

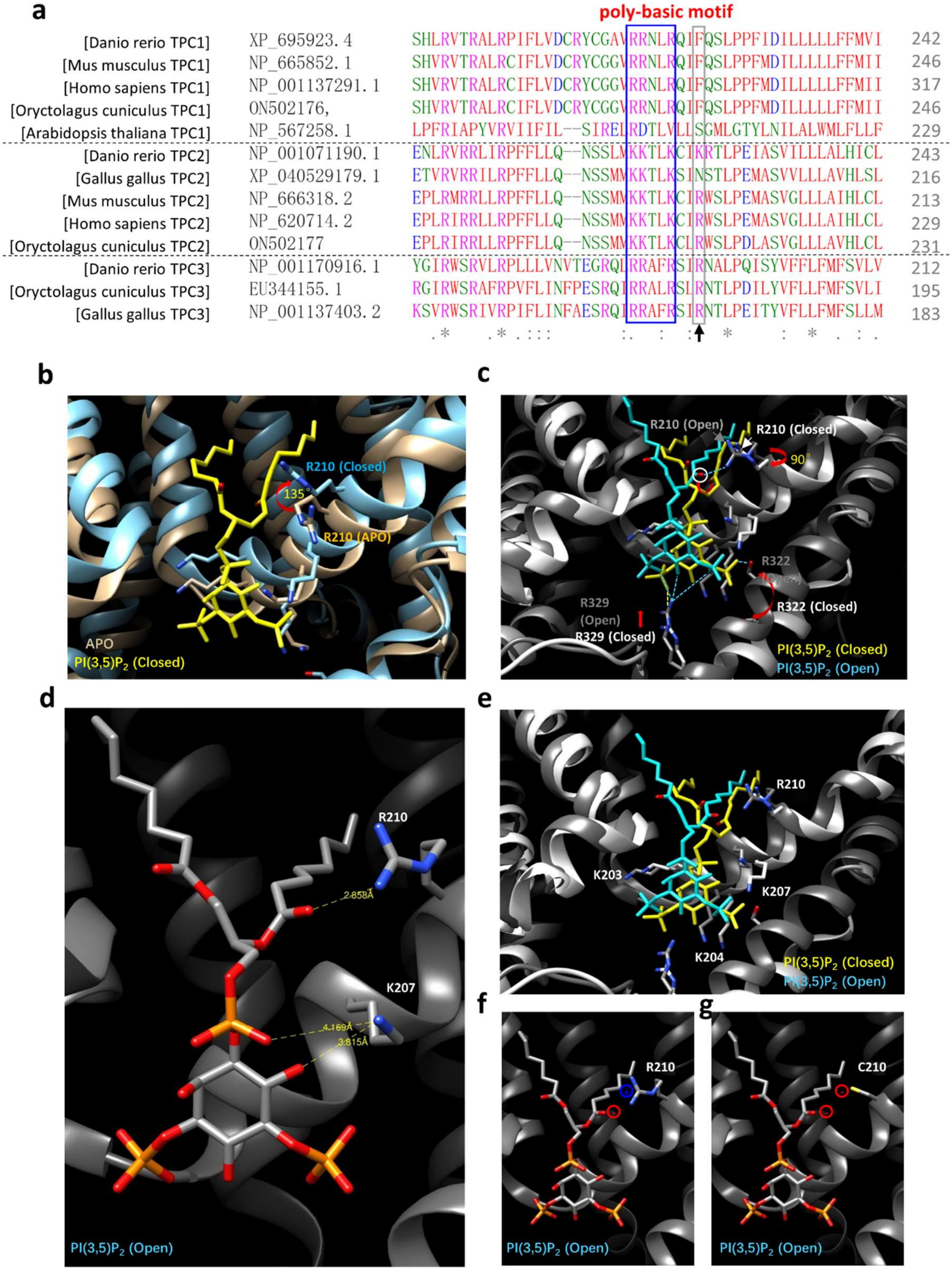
a
poly-basic motif
[Danio rerio TPC1] XP_695923.4 SHLRVTRALRPIFLVDCRYCGAVRRNLRQIFQSLPPFIDILLLLLFFMVI 242
[Mus musculus TPC1] NP_665852.1 SHVRVTRALRCIFLVDCRYCGGVRRNLRQIFQSLPPFMDILLLLLFFMII 246
[Homo sapiens TPC1] NP_001137291.1 SHVRVTRALRCIFLVDCRYCGGVRRNLRQIFQSLPPFMDILLLLLFFMII 317
[Oryctolagus cuniculus TPC1] ON502176, SHVRVTRALRCIFLVDCRYCGGVRRNLRQIFQSLPPFMDILLLLLFFMII 246
[Arabidopsis thaliana TPC1] NP_567258.1 LPFRIAPYVRVIIFIL--SIRELRDTLVLLSGMLGTYLNILALWMLFLLF 229
[Danio rerio TPC2] NP_001071190.1 ENLRVRRLIRPFFLLQ--NSSLMKKTLKCIKRTLPEIASVILLLALHICL 243
[Gallus gallus TPC2] XP_040529179.1 ETVRVRRILRPFFLLQ--NSSMMKKTLKSINSTLPEMASVVLLLAVHLSL 216
[Mus musculus TPC2] NP_666318.2 EPLRMRRLLRPFFLLQ--NSSMMKKTLKCIRWSLPEMASVGLLLAIHLCL 213
[Homo sapiens TPC2] NP_620714.2 EPLRIRRLLRPFFLLQ--NSSMMKKTLKCIRWSLPEMASVGLLLAIHLCL 229
[Oryctolagus cuniculus TPC2] ON502177 EPLRIRRLLRPFFLLQ--NSSMMKKTLKCLRWSLPDLASVGLLLAVHLCL 231
[Danio rerio TPC3] NP_001170916.1 YGIRWSRVLRPLLLVNVTEGRQLRRAFRSIRNALPQISYVFFLFMFSVLV 212
[Oryctolagus cuniculus TPC3] EU344155.1 RGIRWSRAFRPVFLINFPESRQIRRALRSLRNTLPDILYVFLLFMFSVLI 195
[Gallus gallus TPC3] NP_001137403.2 KSVRWSRIVRPIFLINFAESRQIRRAFRSIRNTLPEITYVFLLFMFSLLM 183
.* .*.::: :. : : * : * . : .
b
R210 (Closed)
135°
R210 (APO)
APO
PI(3,5)P2 (Closed)
c
R210 (Open)
R210 (Closed)
90°
R322
R322 (Closed)
R329 (Open)
R329 (Closed)
PI(3,5)P2 (Closed)
PI(3,5)P2 (Open)
d
R210
2.858Å
K207
4.169Å
3.815Å
PI(3,5)P2 (Open)
e
R210
K203
K207
K204
PI(3,5)P2 (Closed)
PI(3,5)P2 (Open)
f
R210
PI(3,5)P2 (Open)
g
C210
PI(3,5)P2 (Open)

Supplementary Fig. 1 Partial sequence alignment of the TPC protein family and structural insights of R210 in TPC2 gating.

(a) Sequence alignment of TPC proteins. The blue box represents a poly-basic motif, and the black arrow indicates the position of R210 in human TPC2 and residues at the equivalent position in TPC2 of other species and TPC paralogs.

(b) Mobilization of R210 side chain when PI(3,5)P_2 (*highlighted in yellow*) binds to TPC2 during the conformational transition from the Apo state (*golden helix and golden-blue guanidinium*) to the ligand-bound closed state (*light blue helix and light-blue and blue guanidinium*), based on the human TPC2 cryo-EM structures. Note that there is a 135° twist of guanidine during the transition, indicating that R210 plays a role in regulating PI(3,5)P_2 binding. Also note that R210 is juxtaposed to the sn-1 fatty acyl chain of PI(3,5)P_2 in the ligand-bound closed state.

(c) Mobilization of R210 side chain and the flip of the PI(3,5)P_2 fatty acyl chains during the conformational transition from the ligand-bound closed state (*light grey*) to ligand-bound open state (*dark grey*). PI(3,5)P_2 is highlighted in yellow in the closed state, but in cyan in the open state. Note that R210 side chain rotated 90° and PI(3,5)P_2 fatty acyl chains flipped to bring the sn-2 carbonyl group (*marked by a white circle*) close to the guanidine group of R210, sufficient to form an electrostatic interaction. R329 and S322 on IS6 helix move toward PI(3,5)P_2 and establish salt bridges and hydrogen bond with the phosphates on the head of PI(3,5)P_2 (*shown in blue dot line*) to open the channel.

(d) Measured distance between the R210 side chain and the carbonyl groups on the fatty acyl chain of PI(3,5)P_2. In the ligand-bound closed state, the distance is 4.668 Å from sn-1 carbonyl group. In the ligand-bound open state, this distance is shortened to 2.858 Å from sn-2 carbonyl group. The closer distance increases stronger electrostatic interaction by ~2.66 folds according to the Coulomb's law.

(e) PI(3,5)P_2 interaction with TPC2 at the open state, highlighting the electrostatic interaction between R210 guanidine group and the sn-2 carbonyl group of PI(3,5)P_2, as well as the hydrophobic interaction between the fatty acyl chains and the S3 and S4 TM segments of TPC2.

(f, g) Electrostatic property change from R210 to C210, which represents that a positive-to-negative attractive force is changed into a negative-to-negative repulsive force, supporting our **Attraction-Repulsion (AR) Model** to explain the profound influence of PI(3,5)P_2-regulated TPC2 gating.

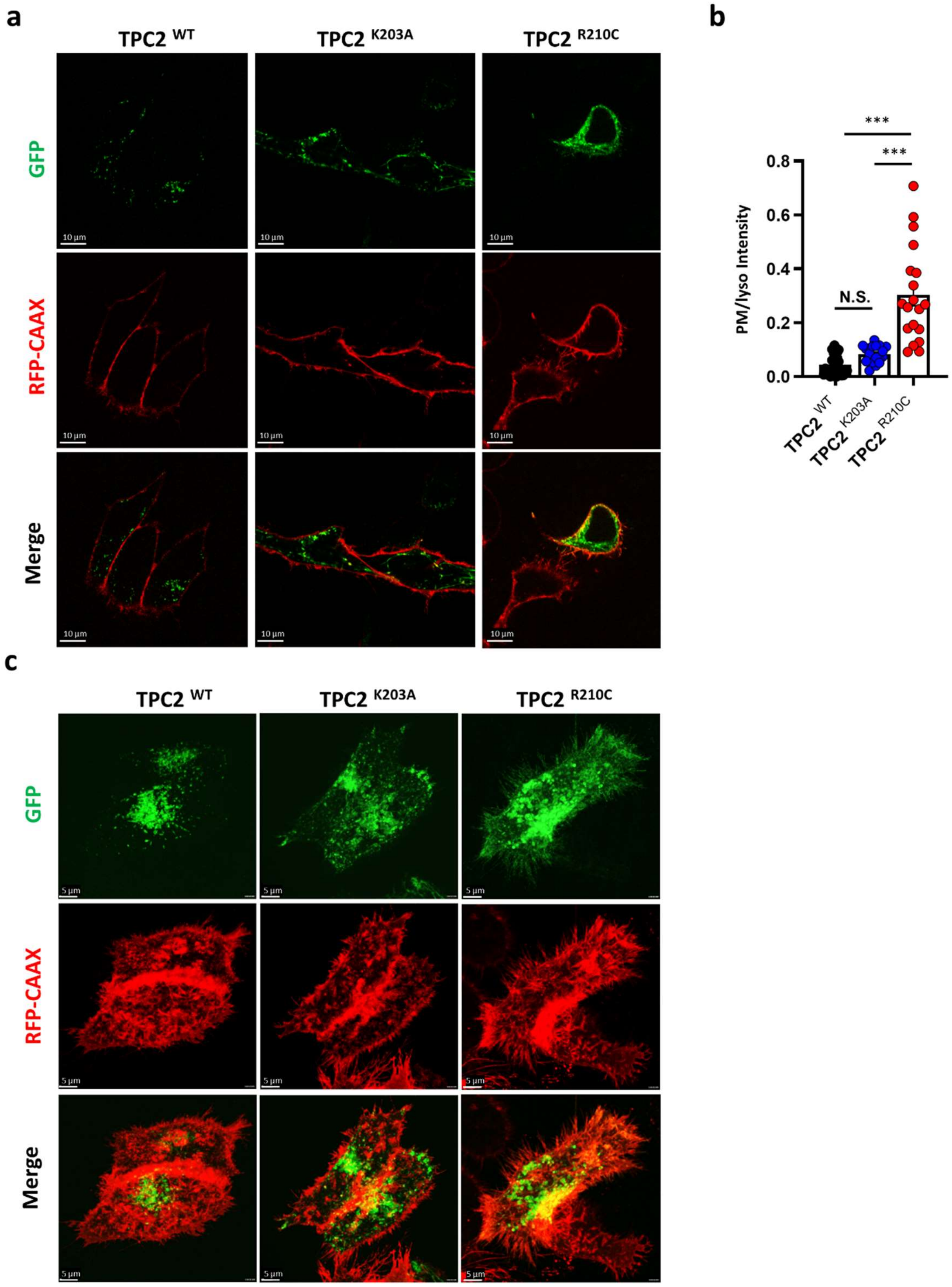
a
TPC2 WT
TPC2 K203A
TPC2 R210C
GFP
RFP-CAAX
Merge
10 µm
b
***
***
N.S.
PM/lyso Intensity
0.8
0.6
0.4
0.2
0.0
TPC2 WT
TPC2 K203A
TPC2 R210C
c
TPC2 WT
TPC2 K203A
TPC2 R210C
GFP
RFP-CAAX
Merge
5 µm

Supplementary Fig. 2 Altered localization and enlarged vacuoles in TPC2^{R210C} overexpressed cells.

(a) Representative confocal images of Hela cells co-transfected with EGFP-TPC2 and RFP-CAAX. Overexpression of TPC2^{R210C} made cells round up and the EGFP signal was partially localized at the PM compared to TPC2^{WT} and TPC2^{K203A}. Scale bar, 10 μm.

(b) Statistics ratio of EGFP fluorescent intensity on the PM to that associated with lysosomes. The ratios (Means ± SEM) are: TPC2^{WT}, 0.04 ± 0.01 (n = 27); TPC2^{K203A}, 0.08 ± 0.01 (n = 19), and TPC2^{R210C}, 0.30 ± 0.04 (n = 19). ∗∗∗$p < 0.001$, N.S., not significant, by one-way ANOVA. Source data are provided as a Source Data file.

(c) 3D scanning images of Hela cells co-transfected with EGFP-TPC2 and RFP-CAAX. Cells overexpressing TPC2^{R210C} exhibited a considerable amount of green fluorescence that co-localized with RFP-CAAX in the pseudopods. Scale bar, 5 μm. This assay was repeated 3 times.

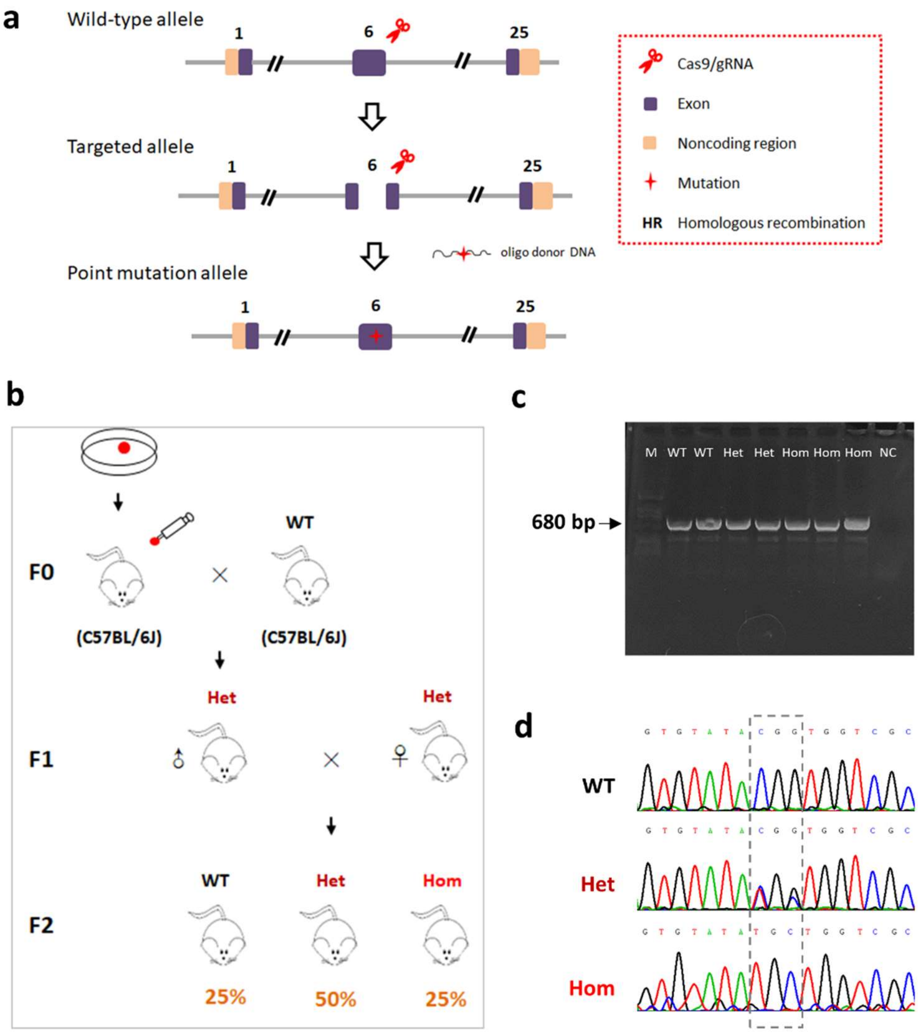

Supplementary Fig 3 Generation of *Tpcn2* R194C knock-in mouse strain with CRISPR-Cas9 editing strategy.

(a) A schematic flowchart for the generation of the mouse knock-in R194C variant with a gRNA targeting exon 6 of *Tpcn2* on the C57BL/6J background.

(b) Mouse mating processes. The mouse obtained from microinjection was mated with C57BL/6J to produce F1 offspring carrying heterozygous knock-in variant. The F1 mice were mated to give rise to F2 offspring.

(c) PCR amplicon product of 680 bp was confirmed on a 1% agarose gel and used for sequencing to conduct genotyping. This assay was repeated twice.

(d) The genotypes of offspring were verified by Sanger sequencing. The dashed grey box indicates the codon site of R194C variant.
