# Supplementary material for: A gain-of-function TPC2 variant R210C increases affinity to PI(3,5)P2 and causes lysosome acidification and hypopigmentation
Source: Nat Commun. 2023 Jan 14;14:226. doi: 10.1038/s41467-023-35786-9 (PMC9840614; doi:10.1038/s41467-023-35786-9)
Supplement: Supplementary file 3 — Reporting Summary [file 41467_2023_35786_MOESM3_ESM.pdf]

nature portfolio

Corresponding author(s): Aihua Wei & Wei Li

Last updated by author(s): Nov 10, 2022

Reporting Summary

Nature Portfolio wishes to improve the reproducibility of the work that we publish. This form provides structure for consistency and transparency in reporting. For further information on Nature Portfolio policies, see our Editorial Policies and the Editorial Policy Checklist.

Statistics

For all statistical analyses, confirm that the following items are present in the figure legend, table legend, main text, or Methods section.

n/a	Confirmed	
☐	☒	The exact sample size (*n*) for each experimental group/condition, given as a discrete number and unit of measurement
☐	☒	A statement on whether measurements were taken from distinct samples or whether the same sample was measured repeatedly
☐	☒	The statistical test(s) used AND whether they are one- or two-sided<br>*Only common tests should be described solely by name; describe more complex techniques in the Methods section.*
☒	☐	A description of all covariates tested
☐	☒	A description of any assumptions or corrections, such as tests of normality and adjustment for multiple comparisons
☐	☒	A full description of the statistical parameters including central tendency (e.g. means) or other basic estimates (e.g. regression coefficient) AND variation (e.g. standard deviation) or associated estimates of uncertainty (e.g. confidence intervals)
☐	☒	For null hypothesis testing, the test statistic (e.g. *F*, *t*, *r*) with confidence intervals, effect sizes, degrees of freedom and *P* value noted<br>*Give P values as exact values whenever suitable.*
☒	☐	For Bayesian analysis, information on the choice of priors and Markov chain Monte Carlo settings
☒	☐	For hierarchical and complex designs, identification of the appropriate level for tests and full reporting of outcomes
☒	☐	Estimates of effect sizes (e.g. Cohen's *d*, Pearson's *r*), indicating how they were calculated

Our web collection on statistics for biologists contains articles on many of the points above.

Software and code

Policy information about availability of computer code

Data collection	ZEN Blue 2.3 SP1 software (Carl Zeiss), pCLAMP 10(Axon instruments), NIS-Elments 4.30.00(Nikon)
Data analysis	pCLAMP 10(Axon instruments), Clampfit 10.4(Molecular Devices),NIS-Elements Viewer(5.21),Chimera(1.16),GraphPad Prizm (7.0), GraphPad Prizm (9.0) , Origin(8.0), Image J(1.53o), Microsoft Excel (2021)

For manuscripts utilizing custom algorithms or software that are central to the research but not yet described in published literature, software must be made available to editors and reviewers. We strongly encourage code deposition in a community repository (e.g. GitHub). See the Nature Portfolio guidelines for submitting code & software for further information.

Data

Policy information about availability of data

All manuscripts must include a data availability statement. This statement should provide the following information, where applicable:

- Accession codes, unique identifiers, or web links for publicly available datasets
- A description of any restrictions on data availability
- For clinical datasets or third party data, please ensure that the statement adheres to our policy

All data supporting the findings of this study and unique biological materials used in this study are available from the corresponding authors upon reasonable request. The source data underlying Figs. 1~8 and Supplementary Fig. 2 are provided as a Source Data file. The structures of TPC2 were retrieved from the PDB database (https://www.rcsb.org/structure) for the Apo state (PDB# 6NQ1), ligand-bound closed state (PDB# 6NQ2), and the ligand-bound open state (PDB# 6NQ0).

Human research participants

Policy information about studies involving human research participants and Sex and Gender in Research.

Reporting on sex and gender	The index patient was a 7-years-old girl. She was diagnosed as albinism according to "Clinical Practice Guidelines for Albinism". Her biological father and mother were also recruited in this study.
Population characteristics	This work does not take into account the covariate-relevant population. Information about studies involving participants is described above.
Recruitment	The proband (with albinism) and her parents visited our out-patient clinic. There is no potential for self-selection bias or other biases.
Ethics oversight	This study was approved by the ethics committees of Beijing Children's Hospital and Beijing Tongren Hospital, Capital Medical University.

Note that full information on the approval of the study protocol must also be provided in the manuscript.

Field-specific reporting

Please select the one below that is the best fit for your research. If you are not sure, read the appropriate sections before making your selection.

☒ Life sciences ☐ Behavioural & social sciences ☐ Ecological, evolutionary & environmental sciences

For a reference copy of the document with all sections, see nature.com/documents/nr-reporting-summary-flat.pdf

Life sciences study design

All studies must disclose on these points even when the disclosure is negative.

Sample size	The sample size is large enough to generate statistical differences(p[illegible]0.05), in order to quantitatively analyze the differences between groups. To demonstrate the scientific of the study design, three or more biological replicates were performed to confirm the conclusions.
Data exclusions	No data were excluded from analyses.
Replication	All measurements were taken from distinct samples, repeated more than three times. All attempts at replication were successful.
Randomization	This experiment is not a randomized controlled experiment, only one trio-family was recruited, so randomization is not relevant to our study.
Blinding	TAll measurements of embryo were divided into groups by genotypes. Two investigators were blinded to group allocation during data collection and/or analysis.

Reporting for specific materials, systems and methods

We require information from authors about some types of materials, experimental systems and methods used in many studies. Here, indicate whether each material, system or method listed is relevant to your study. If you are not sure if a list item applies to your research, read the appropriate section before selecting a response.

Materials & experimental systems

n/a	Involved in the study
☒	☐ Antibodies
☐	☒ Eukaryotic cell lines
☒	☐ Palaeontology and archaeology
☐	☒ Animals and other organisms
☐	☒ Clinical data
☒	☐ Dual use research of concern

Methods

n/a	Involved in the study
☒	☐ ChIP-seq
☒	☐ Flow cytometry
☒	☐ MRI-based neuroimaging

Eukaryotic cell lines

Policy information about cell lines and Sex and Gender in Research

Cell line source(s)	Commercial sources of HEK293 (CAT# CTCC-003-0015) and Hela (CAT# CTCC-001-0006) cell lines were used.
Authentication	None of the cell lines used were authenticated.
Mycoplasma contamination	Both cell lines were purchased with mycoplasma free. The reagent for anti-mycoplasma is routinely included into cell culture, and is routinely tested negative for mycoplasma.
Commonly misidentified lines (See ICLAC register)	No commonly misidentified cell lines was used.

Animals and other research organisms

Policy information about studies involving animals; ARRIVE guidelines recommended for reporting animal research, and Sex and Gender in Research

Laboratory animals	C57BL/6J mice aged 3-6 months were photographed for hair pigment and tail color. Primary mouse embryonic fibroblasts (MEFs) were isolated from E13.5-E14.5 mouse embryonic fibroblasts. Mice are kept in independent ventilated cages in suitable housing conditions as follows, with ambient temperature maintained at 20 ~ 26?, relative humidity at 50% ~ 60%. The dark/light cycle was 12 h/12 h, and light intensity at 15 ~ 20 lx.
Wild animals	The study did not involve wild animals.
Reporting on sex	Sex information has not been collected. Because the Cytogenetic location of TPCN2 gene is 11q13.3, sex-based analysis makes no sense.
Field-collected samples	The study did not involve samples collected from the field.
Ethics oversight	This study was approved by the ethics committees of Beijing Children's Hospital and Beijing Tongren Hospital, Capital Medical University.

Note that full information on the approval of the study protocol must also be provided in the manuscript.

Clinical data

Policy information about clinical studies
All manuscripts should comply with the ICMJE guidelines for publication of clinical research and a completed CONSORT checklist must be included with all submissions.

Clinical trial registration	*Provide the trial registration number from ClinicalTrials.gov or an equivalent agency.*
Study protocol	*Note where the full trial protocol can be accessed OR if not available, explain why.*
Data collection	*Describe the settings and locales of data collection, noting the time periods of recruitment and data collection.*
Outcomes	*Describe how you pre-defined primary and secondary outcome measures and how you assessed these measures.*
